# Teaching an Old Dog New Tricks: Strategies That Improve Early Recognition in Similarity-Based Virtual Screening

**DOI:** 10.3389/fchem.2019.00701

**Published:** 2019-10-23

**Authors:** Ruifeng Liu, Mohamed Diwan M. AbdulHameed, Anders Wallqvist

**Affiliations:** ^1^Department of Defense Biotechnology High Performance Computing Software Applications Institute, Telemedicine and Advanced Technology Research Center, U.S. Army Medical Research and Development Command, Fort Detrick, MD, United States; ^2^The Henry M. Jackson Foundation for the Advancement of Military Medicine, Inc., Bethesda, MD, United States

**Keywords:** lead discovery, virtual screening, early recognition, Tanimoto similarity, z-score, BEDROC, ROCS

## Abstract

High throughput screening (HTS) is an important component of lead discovery, with virtual screening playing an increasingly important role. Both methods typically suffer from lack of sensitivity and specificity against their true biological targets. With ever-increasing screening libraries and virtual compound collections, it is now feasible to conduct follow-up experimental testing on only a small fraction of hits. In this context, advances in virtual screening that achieve enrichment of true actives among top-ranked compounds (“early recognition”) and, hence, reduce the number of hits to test, are highly desirable. The standard ligand-based virtual screening method for large compound libraries uses a molecular similarity search method that ranks the likelihood of a compound to be active against a drug target by its highest Tanimoto similarity to known active compounds. This approach assumes that the distributions of Tanimoto similarity values to all active compounds are identical (i.e., same mean and standard deviation)—an assumption shown to be invalid (Baldi and Nasr, 2010). Here, we introduce two methods that improve early recognition of actives by exploiting similarity information of all molecules. The first method ranks a compound by its highest z-score instead of its highest Tanimoto similarity, and the second by an aggregated score calculated from its Tanimoto similarity values to all known actives and inactives (or a large number of structurally diverse molecules when information on inactives is unavailable). Our evaluations, which use datasets of over 20 HTS campaigns downloaded from PubChem, indicate that compared to the conventional approach, both methods achieve a ~10% higher Boltzmann-enhanced discrimination of receiver operating characteristic (BEDROC) score—a metric of early recognition. Given the increasing use of virtual screening in early lead discovery, these methods provide straightforward means to enhance early recognition.

## Introduction

Lead discovery by high throughput screening (HTS) is often described as a process akin to finding a needle in a haystack (Aherne et al., 2002). Given the significant achievements in automation, major pharmaceutical companies now routinely screen hundreds of thousands of samples to identify compounds that are active against specific drug targets. However, the number of chemicals available for bioactivity testing has increased exponentially over the past decade. For instance, as of 2015, the number of structurally unique chemicals registered in PubChem was more than 60 million (Kim et al., 2016), and in 2018, the total number of organic and inorganic substances disclosed in the literature was estimated to be 154 million^1^. Thus, despite the increased screening capacity, it remains impractical to assay a significant fraction of all available chemicals. Consequently, virtual screening is becoming increasingly important to prioritize and select compounds (Kar and Roy, 2013). The most widely used virtual screening methods are based on molecular similarity searches (Kristensen et al., 2013). These approaches typically rank molecules in a chemical library based on their structural similarity to a set of molecules known to be active against a desired target. Chemicals ranked high on the list can then be acquired and tested for the desired activity or property.

The most commonly used metric to compare the performance of different virtual screening methods is the area under the receiver operating characteristic curve (ROC_AUC) (Triballeau et al., 2005). This is useful for comparing overall performance of methods for ranking a database (Truchon and Bayly, 2007; Zhao et al., 2009). However, the ROC_AUC is inappropriate for virtual screening when the goal is to create a smaller subset enriched with the maximum number of actives (Truchon and Bayly, 2007). The distinction is critical, especially when the chemical libraries are large and only a small fraction of compounds can be tested. Truchon and Bayly (2007) illustrated the difference using three basic cases: (1) half of the actives ranked at the top of a rank-ordered list and the other half at the bottom; (2) all actives randomly distributed across ranks; and (3) all actives ranked in the middle of the list. In all three cases, the ROC_AUC value is 0.5 and, therefore, according to this metric, all three virtual screening methods that generated the three rank-ordered lists perform equally. However, because only a small fraction of chemicals in a large library can be tested, “early recognition” of actives is practically important. That is, case 1 is preferable to case 2 or 3, and case 2 could be considered more desirable than case 3.

Many metrics have been proposed to address early recognition. Examples include the partial area under the ROC curve (McClish, 1989), enrichment factor (Halgren et al., 2004), area under the accumulation curve (Kairys et al., 2006), robust initial enhancement (Sheridan et al., 2001), Boltzmann-enhanced discrimination of the receiver operating characteristic (BEDROC) (Truchon and Bayly, 2007), and predictiveness curve (Empereur-Mot et al., 2015). Although no metric is perfect, perhaps the most frequently adopted is BEDROC, which employs an adjustable parameter, α, to define “early detection.” Truchon and Bayly suggest setting this parameter to 20.0, which dictates that 80% of the maximum contribution to BEDROC comes from the top 8% of the ranked list. A comparatively higher BEDROC score between two virtual screening methods indicates an enhanced ability to enrich the list of top-ranking compounds with active molecules.

Using both AUC_ROC and BEDROC, Nasr et al. (2009) carried out a large-scale study of the performance of 14 similarity search methods, including eight parameter-free methods (no parameters to be learned from training data) and six with one or two parameters to be learned from training data. Consistent with previous results, they found that the best parameter-free method is the Max-Sim method, which ranks molecules based on their maximum Tanimoto coefficient (TC, also commonly referred to as Tanimoto similarity) to the active query molecules. Among the six methods that require parameters to be fit to the data, the exponential Tanimoto discriminant (ETD) method was the best performer overall. This method is defined by the following equations.

(1) $$\begin{array}{rcl} S\left(B\right) & = & \frac{\sum_{i=1}^{m}S\left(A_{i},\;B\right)}{\sum_{j=1}^{n}S\left(I_{j},\;B\right)} \end{array}$$

(2) $$S\left(A,B\right)\;={\left[\lambda^{TC_{AB}}{\left(1-\lambda \right)}^{1-TC_{AB}}\right]}^{\frac{1}{k}}$$

(3) $$\begin{array}{rcl} TC_{AB} & = & \frac{A\cap B}{A\cup B} \end{array}$$

Here, *S*(*B*) denotes the aggregated score for molecule *B*, *m*, and *n*, respectively denote the numbers of active and inactive query molecules, *A*_*i*_ denotes the *i*th active query molecule, *I*_*j*_ denotes the *j*th inactive molecule, *TC*_*AB*_ denotes the TC between molecules *A* and *B*, λ, and *k* denote parameters to be learned from the data. The higher the aggregated score, the more likely it is that molecule *B* is active. Nasr et al. (2009) provided neither recommended default parameter values for λ and *k*, nor values learned from any of their datasets.

In this article, we introduce two parameter-free similarity search methods that improve the early recognition of actives over the Max-Sim method. Using HTS data, we demonstrate that, on average, the BEDROC values derived from both methods are about 10% higher than those derived from the Max-Sim scoring method.

## Methods and Materials

### Rank by Z-Scores

**Figure 1:**
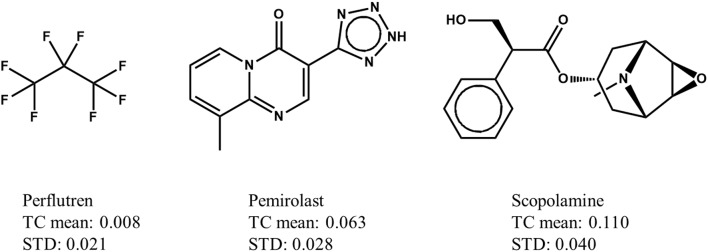
Examples of means and standard deviations (STD) of the Tanimoto coefficients (TCs) of 10,000 compounds randomly selected from the National Cancer Institute's virtual screening library calculated with respect to three drugs approved by the Food and Drug Administration.

In a Max-Sim similarity search, we first calculate all TCs between the compounds in a chemical library and active query molecules. The library compounds are then ranked based on their highest TCs. The underlying assumption is that the higher the TC, the more likely a compound is to be active. This assumption is valid for searches with a single active query molecule, and for searches with multiple active query molecules if the distributions of TCs are identical (i.e., have the same mean and standard deviation irrespective of the query molecules). Although it has been standard practice for many years to conduct Max-Sim similarity searches, no study had examined the statistical distribution of TCs until 2010, when Baldi and Nasr (2010) investigated in detail the significance of Tanimoto similarity. They showed that the statistical distribution of TCs is not invariant, but depends on the number of fingerprint features present in a query molecule. This finding and its implications, however, are largely overlooked by the cheminformatics community, perhaps due to the reasonably good performance of the Max-Sim method and the extremely low mean TCs for any query molecule. As an example, Figure 1 shows the means and standard deviations of the TCs of 10,000 chemicals randomly selected from the U.S. National Cancer Institute (NCI) chemical library calculated with respect to each of three drugs approved by the U.S. Food and Drug Administration. All of the means and standard deviations are close to zero, suggesting that most NCI compounds do not have the same activity as that of the approved drugs. The small mean TCs may obscure an important fact—that the values are not identical and could be significantly different. For instance, the mean TC of scopolamine is 43% higher than that of pemirolast. To appreciate the implications of the difference, let us assume that a molecule has TCs of 0.80 and 0.70 calculated with respect to scopolamine and pemirolast, respectively. Based on the Max-Sim method, one would expect the molecule to have activities more similar to those of scopolamine. However, because of the difference in the means and standard deviations, the z-scores of the molecule calculated with respect to scopolamine and pemirolast are 17.3 and 22.8, respectively, suggesting that the molecule is more likely to have activities similar to those of pemirolast than to those of scopolamine. If we consider that there are differences in mean TCs and standard deviations, then ranking molecules by the maximum z-score is statistically preferable in a similarity search. Accordingly, we designate this approach as the maxZ method.

### Rank by Aggregated Similarity

In the past two decades, HTS has contributed to the discovery of numerous structurally novel active compounds against many important drug targets. As these compounds are identified from large experimentally tested screening libraries, they are classified as either active or inactive based on predefined activity criteria. In follow-up studies based on virtual screening by similarity search methods, only active compounds are used as queries. As noted in the Introduction, Nasr et al. (2009) developed the ETD method, which exploits information of both active and inactive compounds. They found that ETD performed best among 14 parameterized and non-parameterized TC-based similarity search methods. An undesirable feature of this method, however, is that it requires two parameters that may not be universally applicable, but still need to be fit for each individual dataset. Here, we propose an aggregated similarity (AS) method that does not require any parameter fitting based on individual datasets. We define the AS method by the following equation:

(4) $$\begin{array}{r} AS\left(X\right)=\frac{\sum_{i=1}^{m}e^{-\;\frac{1-TC\left(A_{i},X\right)}{\alpha +TC\left(A_{i},X\right)}}}{\sum_{j=1}^{n}e^{-\;\frac{1-TC\left(I_{j},X\right)}{\alpha +TC\left(I_{j},X\right)}}} \end{array}$$

where, *X* denotes a compound in a chemical library, *m* and *n*, respectively denote the number of active and inactive molecules, *TC*(*A*_*i*_, *X*) denotes the TC between the *i*th active molecule and *X*, *TC*(*I*_*j*_, *X*) denotes the TC between the *j*th inactive molecule and *X*, and α is set to 10^−6^—a small number to avoid division by zero when TC equals zero. Possible *AS(X)* values range from zero to infinity, where zero indicates that molecule *X* shares no fingerprint features with any of the active query molecules, i.e., all *TC*(*A*_*i*_, *X*) = 0, and infinity indicates that molecule *X* shares no fingerprint features with any of the inactive query molecules. In reality, because the number of inactive molecules is large (i.e., a positive is like a needle in a haystack and, therefore, most molecules can be classified as inactives), the probability of *X* sharing no fingerprint features with any of the inactive query molecules is zero, unless a very small number of inactive query molecules is used (even though a large number of them should be available).

One problem with using information on inactive compounds is that the results of large-scale screening campaigns are not equally reliable for active and inactive compounds. This is because such campaigns are typically executed in multiple confirmatory steps focusing on active compounds. The first step involves an initial primary screening of a large number of samples at a single concentration with few or no replicates. Samples deemed to meet the primary activity criteria are then selected and retested in multiple replicates, usually with counter-assays to affirm activity. Samples that satisfy the retesting criteria may be further tested at multiple concentrations to determine potency. One consequence of this screening protocol is that the activities of a positive compound are more reliable because they are reassessed in multiple tests, whereas compounds fail to meet primary active criteria are not retested to confirm inactivity. As a result, the set of inactive molecules is likely to contain false negatives. A more obvious problem with the AS method is that it cannot be applied to cases where information on inactives is unavailable. As a means to overcome this challenge, we suggest that a set of structurally diverse compounds can be used as putative inactive compounds. This is because compounds that are truly active against the most valuable drug targets are rare (i.e., needles in a haystack). Therefore, within a structurally diverse set of compounds, the number of compounds that are active against a drug target should be small. Here, we tested the validity of this hypothesis by using 10,000 structurally diverse compounds as putative inactives. We selected these compounds by clustering ~275,000 compounds of the NCI virtual screening library (Shiryaev et al., 2011) into 10,000 clusters based on the TC (a measure of molecular similarity), and selecting the cluster centers as structurally diverse compounds to represent coverage of the chemical space of the full dataset. In doing so, we considered a singleton as a cluster of size one.

### Datasets

We evaluated the performance of the similarity search methods using HTS data generated from the National Center for Advancing Translational Sciences of the National Institutes of Health. We downloaded the data in two batches. The first batch consisted of the results of ~8,000 samples screened against 10 toxicity-related targets using 12 different assays, with two different assays deployed for two of the 10 targets. Thus, roughly the same 8,000 samples were tested in 12 assays, generating 12 molecular activity datasets. As these datasets were used in the Tox21 Data Challenge for molecular activity predictions (Huang and Xia, 2017), we downloaded them from Tox21 Data Challenge web site^2^. Because the datasets were relatively small (consisting of ~7,000 structurally unique compounds), we used them to evaluate maxZ scoring methods based on two-dimensional (2-D) molecular fingerprints and three-dimensional (3-D) molecular shapes.

A library consisting of 8,000 samples can hardly be considered a “large” library for HTS. Therefore, we used a second batch of data that consisted of results for a few thousand to a few hundred thousand samples screened against 12 different molecular targets. We downloaded these data from the PubChem web site (https://pubchem.ncbi.nlm.nih.gov/) using their assay IDs as queries. Table 1 shows the assay IDs of these datasets together with the Tox21 Challenge datasets. Details of the datasets, including the molecular targets, specific assays, number of samples screened, and number of samples deemed active, can be found from PubChem using the respective assay IDs as queries.

**Table 1:** PubChem datasets used in this study to evaluate performance of similarity search methods.

**Dataset^a^**	**PubChem Assay ID^b^**	**Samples^c^**	**Unique structures^d^**	**Unique actives^e^**
AHR	743122	8169	6318	723
AR	743040	9362	7009	290
ARE	743219	7167	5643	889
AR-lbd	743053	8599	6524	233
Aromatase	743139	7226	5601	273
ATAD5	720516	9091	6825	253
ER	743079	7697	5993	716
ER-lbd	743077	8753	6727	324
HSE	743228	8150	6253	337
MMP	720637	7320	5625	885
P53	720552	8634	6544	410
PPARg	743140	8184	6232	174
4-MU	589	59070	58199	6146
ALDH1A1	1030	220365	215450	15847
BRCA1	624202	377534	373883	3938
DNApb	485314	337903	333082	4466
ERK	1454	133383	130623	532
GCN5L2	504327	387577	379179	741
hERG	588834	5363	4568	553
Lucif	411	72335	70939	1558
MiRNAs	2289	336623	332205	3265
Mitoch	485298	322909	320471	734
NPC1	485313	321376	319001	7532
PR901	1347036	9523	7177	111

a *All datasets are derived from quantitative high throughput screening conducted at the National Center for Advancing Translational Sciences to ascertain chemical activities against different molecular targets. The first 12 datasets were used in the 2014 Tox21 Data Challenge*.

b *The datasets can be accessed from the PubChem website using the assay IDs as queries*.

c *Total number of samples screened in each dataset*.

d *Number of structurally unique parent molecules (non-salts, non-mixtures) derived from retaining the largest chemical structure in each sample and performing structure standardization*.

e *Number of structurally unique active parent molecules*.

*Dataset names: AHR, activators of aryl hydrocarbon receptor; AR, activators of androgen receptor; AR-lbd, activators of androgen receptor ligand binding domain; Aromatase, aromatase inhibitors; ER, estrogen receptor activators; ER-lbd, activators of estrogen receptor ligand binding domain; PPARg, activators of peroxisome proliferator-activated receptor gamma; ARE, activators of antioxidant response element; ATAD5, ATPase family AAA domain-containing protein 5; HSE, activators of heat shock response signaling pathway; MMP, disruptors of mitochondrial membrane potential; p53, activators of p53 signaling pathway; hERG, blockers of hERG potassium channel; PR901, agonists of progesterone receptor; 4-MU, spectroscopic response at the 4-methylumbelliferone region as a counter assay for fluorescence detection; Lucif, inhibitors of Luciferase; ERK, inhibitors of mitogen-activated protein kinase 1; ALDH1A1, inhibitors of aldehyde dehydrogenase 1 family, member A1; NPC1, promoters of Niemann-Pick C1 protein precursor; Mitoch, inhibitors of mitochondrial division; MiRNAs, modulators of miRNAs; DNApb, inhibitors of DNA polymerase beta; BRCA1, activators of BRCA1 expression; GCN5L2, inhibitors of histone acetyltransferase KAT2A*.

Because some samples were prepared from the same parent chemicals, we first cleaned the data before using them to evaluate the performance of the similarity search methods. We first removed counter-ions in salts and retained the largest component in samples consisting of non-bonded (i.e., disconnected) components. We then standardized the structures by neutralizing acids and bases (protonating acids and de-protonating bases) and generating a canonical SMILES from the standardized structure for each sample. For the results of each dataset, we applied a first-pass filter on canonical SMILES and retained only the first sample entry of a structurally unique parent compound. Table 1 summarizes the resulting number of structurally unique parent compounds tested and the number of structurally unique actives from each assay.

In addition to the 24 HTS datasets, we also evaluated performance of the methods on 40 datasets in the Directory of Useful Decoys (DUD) (Huang et al., 2006) and an enhanced version of DUD consisting of 102 datasets called DUDE datasets (Mysinger et al., 2012). Each of these datasets consists of compounds known active on a protein target and many compounds of similar physicochemical properties as the actives but of very different molecular structures as the actives. These datasets are designed for evaluating the performance of docking-based virtual screening methods. We expect them to be less challenging than the HTS datasets for 2-D molecular similarity search methods, because in these datasets the actives and decoys are well-separated in molecular structure space and, therefore, any fingerprint-based similarity search methods are expected to perform well on these datasets.

### Evaluation Protocol

To evaluate the performance of the methods, for each dataset we randomly selected 100 actives as the queries, and combined the other actives with the other compounds tested. We then calculated the maximum TC for each of these compounds with respect to the queries, as well as the maximum z-score and AS score. For these calculations, we used the extended connectivity fingerprint (Rogers and Hahn, 2010) with a maximum diameter of four chemical bonds (ECFP_4) and a fixed fingerprint length of 2,048 bits. We calculated ROC_AUC and BEDROC values for the Max-Sim, maxZ, and AS methods. For all BEDROC calculations, we used the default parameter setting of α = 20.0, i.e., corresponding to 80% of the maximum contribution to BEDROC coming from the top 8% of the list of ranked molecules. To ensure statistical significance of the findings, we repeated the calculations nine times, using 100 randomly selected actives as queries each time. We compared the performance of the methods based on the resulting mean ROC_AUC and BEDROC values.

## Results

### Performance of the maxZ Method

Table 2 shows a summary of the mean ROC_AUC and BEDROC values derived from the Max-Sim and maxZ methods for the 24 datasets. The mean ROC_AUC values derived from the Max-Sim method and those derived from the maxZ method were similar, with the latter only 3.7% higher than the former. In contrast, the mean difference in BEDROC values between the maxZ and Max-Sim methods was as high as 15%. However, the result for one dataset, NPC1, was an outlier, as the difference was as high as 170%, and the mean difference in BEDROC values decreased to 8.7% when it was excluded. Nonetheless, the maxZ method still outperformed the Max-Sim method, as the ROC_AUC and BEDROC values derived from maxZ were smaller than those derived from Max-Sim in only two of the 24 datasets. Although the differences between the maxZ and Max-Sim results were small for some datasets, for those showing a considerable difference, maxZ performed significantly better. For instance, the ROC_AUC values derived from maxZ were at least 5% higher than those derived from Max-Sim in 8 of the 24 datasets, whereas Max-Sim performed better than maxZ by 5% or more in only two datasets. This difference was even more pronounced for BEDROC values, as maxZ outperformed Max-Sim by 5% or more in 15 of the 24 datasets, whereas the opposite was true in only one dataset. Overall, the ROC_AUC values show that the maxZ method performs only slightly better than the Max-Sim method for ranking all samples in the dataset, whereas the BEDROC values indicate that the maxZ method performs markedly better than the Max-Sim method in the early recognition of active compounds.

**Table 2:** Mean and standard deviation of ROC_AUC and BEDROC values derived from a similarity search using the rank by maximum similarity (Max-Sim) and maximum z-score (maxZ) approaches over 10 runs, each with 100 randomly selected actives as queries.

	**ROC_AUC**					**BEDROC**				
	**Max-Sim**		**maxZ**			**Max-Sim**		**maxZ**		
**Dataset**	**Mean**	**Std**	**Mean**	**Std**	**%Diff^a^**	**Mean**	**Std**	**Mean**	**Std**	**%Diff^b^**
AHR	0.754	0.011	0.759	0.012	0.7	0.402	0.016	0.365	0.019	−9.3
AR	0.740	0.009	0.748	0.009	1.0	0.482	0.019	0.509	0.014	5.6
ARE	0.539	0.008	0.544	0.009	1.0	0.225	0.014	0.266	0.018	17.9
AR-lbd	0.815	0.011	0.811	0.011	−0.5	0.607	0.023	0.610	0.028	0.6
Aromatase	0.662	0.017	0.686	0.016	3.6	0.263	0.026	0.291	0.019	10.7
ATAD5	0.713	0.022	0.724	0.018	1.6	0.303	0.025	0.313	0.020	3.4
ER	0.665	0.005	0.667	0.009	0.4	0.380	0.009	0.395	0.012	3.8
ER-lbd	0.715	0.011	0.726	0.010	1.5	0.381	0.026	0.382	0.025	0.0
HSE	0.579	0.027	0.595	0.027	2.8	0.186	0.020	0.205	0.023	10.3
MMP	0.694	0.009	0.704	0.018	1.4	0.414	0.024	0.469	0.025	13.2
P53	0.611	0.013	0.649	0.013	6.1	0.251	0.016	0.265	0.012	5.8
PPARg	0.681	0.018	0.686	0.027	0.7	0.274	0.031	0.277	0.030	1.0
4-MU	0.565	0.007	0.604	0.007	7.0	0.272	0.010	0.316	0.009	16.1
ALDH1A1	0.506	0.004	0.513	0.009	1.3	0.104	0.007	0.111	0.007	6.8
BRCA1	0.667	0.006	0.694	0.004	4.2	0.147	0.006	0.155	0.006	5.5
DNApb	0.591	0.011	0.633	0.012	7.1	0.137	0.006	0.163	0.012	18.9
ERK	0.647	0.021	0.698	0.017	8.0	0.235	0.017	0.250	0.016	6.3
GCN5L2	0.541	0.014	0.651	0.016	20.5	0.179	0.015	0.245	0.015	36.9
hERG	0.745	0.009	0.732	0.013	−1.8	0.460	0.009	0.447	0.012	−2.7
Lucif	0.707	0.008	0.737	0.010	4.1	0.255	0.010	0.268	0.012	4.9
MiRNAs	0.574	0.004	0.609	0.010	6.1	0.128	0.004	0.144	0.008	12.0
Mitoch	0.510	0.006	0.546	0.007	7.0	0.079	0.007	0.101	0.006	28.9
NPC1	0.653	0.007	0.696	0.007	6.5	0.079	0.007	0.213	0.006	170.0
PR901	0.931	0.013	0.945	0.013	1.5	0.800	0.025	0.822	0.022	2.8
**Mean**					**3.8**					**15.4**

a *Percent difference between mean ROC_AUC values for Max-Sim and maxZ methods*.

b *Percent difference between mean BEDROC values for Max-Sim and maxZ methods*.

A popular 3-D equivalent of 2-D fingerprint-based molecular similarity search is the Rapid Overlay of Chemical Structures (ROCS) method (OpenEye Scientific Software, Santa Fe, NM) (Fontaine et al., 2007), which calculates the Tanimoto similarity between 3-D molecular shapes and pharmacophore features. Because of the similarity between 2-D fingerprint-based and 3-D ROCS-based similarity searches, we hypothesized that the maxZ method would also improve early recognition for ROCS-based 3-D similarity searches. To test this hypothesis, we generated up to 15 low-energy conformers for each molecule in the 12 datasets used in the 2014 Tox21 Data Challenge, using Omega version 3.0.1.2 (OpenEye Scientific Software) with default parameters (Hawkins et al., 2010). We then conducted ROCS-based similarity searches for each dataset using randomly selected 10% actives as active queries. Each query molecule was represented by up to 15 of its lowest-energy conformers. We calculated the Tanimoto combo similarity (commonly called the combo score, which is the sum of the shape TC and color force field TCs) pairwise between the conformers of each active query and each conformer of the other compounds using ROCS version 3.2.2.2 with default parameters. The maximum Tanimoto combo score between a query molecule and a non-query molecule is designated as the Tanimoto combo score of the non-query molecule. We then calculated the ROC_AUC and BEDROC values using the maximum Tanimoto combo scores and the maximum z-scores calculated from the Tanimoto combo scores. We repeated this calculation nine more times, each with 10% of the actives randomly selected as active query molecules. Table 3 shows the means and standard deviations of the ROC_AUC and BEDROC values. The results were similar to those of 2-D fingerprint-based similarity searches, i.e., the ROC_AUC values derived from the maxZ method were a few percentage points higher than those derived from the Max-Sim method, but the difference between BEDROC values was 12,6% on average. Thus, sorting the samples by the maximum z-values of the combo scores led to a substantial improvement in early recognition.

**Table 3:** Mean and standard deviation of ROC_AUC and BEDROC values derived from a ROCS-based 3-D molecular similarity search using the rank by maximum similarity (Max-Sim) and maximum z-score (maxZ) methods.

	**ROC_AUC**					**BEDROC**				
	**Max-Sim**		**maxZ**			**Max-Sim**		**maxZ**		
**Dataset**	**Mean**	**Std**	**Mean**	**Std**	**%Diff^a^**	**Mean**	**Std**	**Mean**	**Std**	**%Diff^b^**
AHR	0.588	0.004	0.603	0.004	2.5	0.112	0.003	0.126	0.003	12.7
AR	0.729	0.007	0.749	0.015	2.7	0.201	0.010	0.234	0.013	16.2
ARE	0.547	0.004	0.560	0.005	2.5	0.090	0.003	0.101	0.002	12.6
AR-lbd	0.645	0.020	0.661	0.023	2.4	0.168	0.014	0.208	0.012	23.3
Aromatase	0.562	0.011	0.579	0.013	3.1	0.068	0.005	0.080	0.004	17.2
ATAD5	0.663	0.010	0.686	0.012	3.5	0.164	0.009	0.183	0.009	11.5
ER	0.685	0.007	0.703	0.009	2.6	0.168	0.007	0.184	0.005	10.1
ER-lbd	0.679	0.007	0.697	0.008	2.8	0.181	0.006	0.201	0.006	11.0
HSE	0.526	0.011	0.538	0.012	2.3	0.078	0.005	0.085	0.005	8.7
MMP	0.553	0.002	0.568	0.003	2.6	0.119	0.003	0.127	0.001	7.0
P53	0.575	0.009	0.590	0.011	2.5	0.091	0.006	0.101	0.004	11.2
PPARg	0.569	0.016	0.592	0.020	3.9	0.104	0.010	0.114	0.008	9.7
**Mean**					**2.8**					**12.6**

a *Percent difference between mean ROC_AUC values for Max-Sim and maxZ methods*.

b *Percent difference between mean BEDROC values for Max-Sim and maxZ methods*.

We evaluated the maxZ method for 3-D similarity search of the Tox21 Challenge datasets only, because the datasets were small (7,009 structurally unique compounds in the largest) and the computations could be completed within a reasonable amount of time. Most of the other datasets are much larger, with up to a few hundred thousand structurally unique compounds. We did not evaluate the performance of the maxZ method on these datasets, because the ROCS calculations would have required substantially more computing resources.

### Performance of the AS Method

Table 4 shows the ROC_AUC and BEDROC values calculated from the Max-Sim and AS methods for the 12 Tox21 Challenges datasets. We calculated the AS score using a negative set (inactives) of 1,000 randomly selected compounds from the set of all screening negatives. We used the rest of the actives and inactives in each dataset as test data to evaluate the performance of each similarity search method. Both ROC_AUC and BEDROC values calculated from AS scores were significantly higher than the corresponding values obtained using the Max-Sim method, confirming that exploiting the available information on inactives improves the performance of both virtual screening methods. Note that the improvement of BEDROC values is significantly greater than that of ROC_AUC values, suggesting that the performance gains are mainly due to early recognition of actives in the AS method.

**Table 4:** Mean and standard deviation of ROC_AUC and BEDROC values derived from a fingerprint-based similarity search using the rank by maximum similarity (Max-Sim) and rank by aggregated score (AS) methods.

	**ROC_AUC**					**BEDROC**				
	**Max-Sim**		**AS**			**Max-Sim**		**AS**		
**Dataset**	**Mean**	**Std**	**Mean**	**Std**	**%Diff^a^**	**Mean**	**Std**	**Mean**	**Std**	**%Diff^b^**
AHR	0.734	0.010	0.829	0.010	13.0	0.385	0.016	0.534	0.018	38.6
AR	0.749	0.013	0.809	0.013	8.0	0.443	0.020	0.618	0.019	39.7
ARE	0.537	0.008	0.650	0.011	21.0	0.264	0.021	0.404	0.015	53.2
AR-lbd	0.795	0.017	0.823	0.014	3.5	0.574	0.026	0.605	0.022	5.5
Aromatase	0.615	0.016	0.727	0.021	18.2	0.219	0.029	0.323	0.019	47.3
ATAD5	0.652	0.018	0.717	0.018	9.9	0.273	0.023	0.303	0.024	11.0
ER	0.602	0.024	0.683	0.024	13.6	0.234	0.016	0.439	0.039	87.7
ER-lbd	0.689	0.014	0.713	0.011	3.6	0.374	0.027	0.386	0.017	3.3
HSE	0.557	0.009	0.650	0.012	16.8	0.122	0.010	0.210	0.024	71.8
MMP	0.663	0.011	0.760	0.007	14.6	0.398	0.030	0.565	0.026	41.9
P53	0.588	0.020	0.723	0.013	23.0	0.224	0.017	0.262	0.020	16.9
PPARg	0.678	0.029	0.744	0.025	9.7	0.262	0.036	0.279	0.036	6.3
**Mean**					**12.9**					**35.3**

a *Percent difference between mean ROC_AUC values for Max-Sim and AS methods*.

b *Percent difference between mean BEDROC values for Max-Sim and AS methods*.

As noted in section Rank by Aggregated Similarity, because drug discovery involves rigorous confirmation of the activities of actives, but rarely any investments in efforts to confirm inactivity, information on inactive compounds is usually less reliable than that on active compounds. In addition, for some projects, active queries are not derived from screening of chemical libraries and, hence, there is no information on inactive compounds. However, because the number of compounds that are active against any drug target can be assumed to be miniscule compared to the number of all available compounds, we hypothesized that a large number of structurally diverse compounds should be able to serve as putative inactive compounds for the AS method. To test this hypothesis, we repeated the evaluation above, using 10,000 structurally diverse compounds selected from the NCI library. Table 5 shows that the replacement of inactive compounds by structurally diverse compounds led to a significant performance deterioration of the AS method, especially in terms of ROC_AUC values, which were only 2.6% higher on average than those of the Max-Sim method. However, the overall mean BEDROC value was still 14.6% higher than that of the Max-Sim method, indicating that early recognition improved even when inactive compounds from screening were unavailable.

**Table 5:** Mean and standard deviation of ROC_AUC and BEDROC values derived from a fingerprint-based similarity search using the rank by maximum similarity (Max-Sim) and rank by aggregated score (AS) methods, using 10,000 structurally diverse compounds as inactive compounds.

	**ROC_AUC**					**BEDROC**				
	**Max-Sim**		**AS**			**Max-Sim**		**AS**		
**Dataset**	**Mean**	**Std**	**Mean**	**Std**	**%Diff^a^**	**Mean**	**Std**	**Mean**	**Std**	**%Diff^b^**
AHR	0.730	0.010	0.758	0.009	3.8	0.337	0.023	0.407	0.017	20.8
AR	0.754	0.012	0.768	0.011	1.9	0.436	0.024	0.596	0.017	36.8
ARE	0.535	0.007	0.549	0.009	2.6	0.224	0.021	0.258	0.024	15.2
AR-lbd	0.790	0.018	0.807	0.021	2.2	0.550	0.020	0.614	0.030	11.6
Aromatase	0.621	0.024	0.663	0.023	6.7	0.202	0.040	0.289	0.036	43.2
ATAD5	0.653	0.024	0.666	0.025	2.0	0.268	0.021	0.281	0.023	4.9
ER	0.600	0.009	0.567	0.014	−5.5	0.197	0.010	0.136	0.021	−30.8
ER-lbd	0.683	0.018	0.712	0.017	4.2	0.366	0.030	0.461	0.019	25.8
HSE	0.553	0.013	0.576	0.016	4.2	0.118	0.018	0.147	0.026	24.4
MMP	0.651	0.016	0.689	0.012	5.9	0.336	0.029	0.454	0.019	35.0
P53	0.573	0.018	0.594	0.020	3.6	0.203	0.026	0.202	0.020	−0.2
PPARg	0.689	0.023	0.689	0.022	0.0	0.278	0.024	0.245	0.043	−12.0
**Mean**					**2.6**					**14.6**

a *Percent difference between mean ROC_AUC values for the Max-Sim and AS methods*.

b *Percent difference between mean BEDROC values for the Max-Sim and AS methods*.

To assess the validity of the findings on the 12 Tox21 Challenge datasets for a wider range of datasets with the number of chemicals ranging from a few thousand to few hundred thousand, we conducted virtual screening using the AS method and the same 10,000 structurally diverse NCI compounds as putative inactive compounds. The results were comparable to those obtained from the Tox21 Challenge datasets, indicating that the method is applicable to a wide range of HTS datasets (Table 6).

**Table 6:** Mean and standard deviation of ROC_AUC and BEDROC values derived from a fingerprint-based similarity search using the rank by maximum similarity (Max-Sim) and rank by aggregated score (AS) methods, using 10,000 structurally diverse compounds as inactive compounds.

	**ROC_AUC**					**BEDROC**				
	**Max-Sim**		**AS**			**Max-Sim**		**AS**		
**Dataset**	**Mean**	**Std**	**Mean**	**Std**	**%Diff^a^**	**Mean**	**Std**	**Mean**	**Std**	**%Diff^b^**
4-MU	0.513	0.007	0.525	0.016	2.3	0.143	0.012	0.175	0.014	22.1
ALDH1A1	0.510	0.004	0.519	0.004	1.8	0.142	0.004	0.144	0.003	1.0
BRCA1	0.643	0.008	0.657	0.008	2.3	0.143	0.008	0.146	0.009	2.1
DNApb	0.527	0.010	0.588	0.009	11.7	0.111	0.007	0.173	0.007	55.5
ERK	0.624	0.011	0.672	0.008	7.6	0.247	0.014	0.299	0.011	21.3
GCN5L2	0.542	0.015	0.542	0.017	−0.1	0.132	0.009	0.133	0.012	0.9
hERG	0.725	0.011	0.794	0.006	9.6	0.411	0.032	0.522	0.024	27.2
Lucif	0.735	0.009	0.782	0.006	6.3	0.285	0.015	0.335	0.010	17.5
MiRNAs	0.613	0.006	0.635	0.006	3.6	0.143	0.006	0.153	0.007	7.0
Mitoch	0.512	0.008	0.513	0.007	0.1	0.082	0.008	0.097	0.008	18.4
NPC1	0.681	0.005	0.705	0.007	3.6	0.222	0.005	0.242	0.008	9.0
PR901	0.896	0.019	0.901	0.020	0.5	0.718	0.030	0.757	0.037	5.5
**Mean**					**4.1**					**15.6**

a *Percent difference between mean ROC_AUC values for the Max-Sim and AS methods*.

b *Percent difference between mean BEDROC values for the Max-Sim and AS methods*.

### Performance of the maxZ and AS Methods on the DUD and DUDE Datasets

Because most DUD and DUDE datasets contain <100 actives, we performed evaluations on these datasets by randomly selecting 10% of the actives as queries. We used the remaining actives and all decoys as test sets to evaluate the performance of the methods. As these datasets do not contain any experimentally determined inactives, we used the same set of 10,000 structurally diverse NCI compounds as putative inactives in testing the performance of the AS method. Table S1 shows detailed results obtained from the 40 DUD and 102 DUDE datasets and Table 7 summarizes these results.

**Table 7:** Summary of the performance of similarity search methods on 40 DUD^a^ and 102 DUDE^b^ datasets.

**Metric**	**Mean Max-Sim value^c^**	**Mean%diff^d^**	**Diff ≥ 0%^e^**	**Diff < 0%^f^**	**Diff ≥ 1%^g^**	**Diff < −1%^h^**
**MaxZ vs. Max-Sim method on 40 DUD datasets**						
ROC_AUC	0.91	1.0	30	10	13	0
BEDROC	0.79	1.7	30	10	16	1
**AS vs. Max-Sim method on 40 DUD datasets**						
ROC_AUC	0.90	1.2	28	12	15	3
BEDROC	0.76	1.1	25	15	16	10
**MaxZ vs. Max-Sim method on 102 DUDE datasets**						
ROC_AUC	0.96	0.5	90	12	18	0
BEDROC	0.90	0.7	87	15	28	2
**AS vs. Max-Sim method on 102 DUD datasets**						
ROC_AUC	0.96	0.2	55	47	13	3
BEDROC	0.90	0.2	58	44	27	20

a *DUD: Directory of Useful Decoys, http://dud.docking.org/*.

b *DUDE: Database of Useful Decoys: Enhanced, http://dude.docking.org/*.

c *Mean ROC_AUC or BEDROC value calculated from the Max-Sim method over 40 DUD or 102 DUDE datasets*.

d *Mean percentage difference between ROC_AUC or BEDROC values derived from the maxZ or AS methods and the Max-Sim method*.

e *Number of datasets for which ROC_AUC or BEDROC values calculated from the maxZ or AS methods were higher than or equal to the corresponding values calculated from the Max-Sim method, i.e., the number of datasets on which the maxZ or AS method performed comparable to or better than the Max-Sim method did*.

f *Number of datasets for which ROC_AUC or BEDROC values calculated from the maxZ or AS methods were lower than the corresponding values calculated from the Max-Sim method, i.e., the number of datasets on which the maxZ or AS method performed worse than the Max-Sim method*.

g *Number of datasets for which ROC_AUC or BEDROC values calculated from the maxZ or AS methods were at least 1% higher than the corresponding values calculated from the Max-Sim method*.

h *Number of datasets for which ROC_AUC or BEDROC values calculated from the Max-Sim method was more than 1% higher than the corresponding values calculated from the maxZ or AS methods*.

The most obvious difference between the summary results in Table 7 and the results in Table 2 was that the ROC_AUC and BEDROC values of the HTS datasets derived from the Max-Sim method were significantly lower than the corresponding values of the DUD and DUDE datasets. The mean ROC_AUC values of the DUD and DUDE datasets were 0.90 and 0.96, respectively, and the corresponding mean BEDROC values were 0.76 and 0.90. These values were significantly higher than the corresponding mean ROC_AUC and BEDROC values of 0.66 and 0.29 for the 24 HTS datasets. These results corroborate our expectation that, because the actives and decoys are well-separated in molecular structure space, the DUD and DUDE datasets present much less of a challenge than do the HTS datasets for similarity search methods. Because the Max-Sim method achieved near perfect performance for these datasets, as indicated by an average ROC_AUC value of 0.96 and an average BEDROC value of 0.90 for the DUDE datasets, any improvement beyond the Max-Sim results will necessarily be small given the little room left for improvement. Indeed, Table 7 shows that on average, the ROC_AUC or BEDROC values derived from the maxZ or AS method were only about 1% higher and <1% higher than the corresponding values of the Max-Sim method for the DUD and DUDE datasets, respectively. Nevertheless, Table 7 shows that the number of datasets for which the maxZ and AS methods performed better than the Max-Sim method by more than 1% was significantly higher than that for which the Max-Sim method performed better by more than 1%. Thus, for these datasets, the maxS and AS methods still outperformed the Max-Sim method (albeit with a smaller effect size) even though the Max-Sim method already achieved near perfect performance.

## Discussion

Fingerprint-based molecular similarity search is one of the most important tools for virtual screening of large chemical libraries. Over the years, many similarity search methods have been investigated, but the simple, parameter-free, rank-by-maximum Tanimoto similarity approach remains a popular method. It achieves robust performance based on the Tanimoto similarity of each compound in a compound library to its closest query molecule and disregarding its similarity to all other query molecules. In addition, it compares the values of Tanimoto similarity to different query molecules directly. This is theoretically correct only if the distribution of similarity values to all other query molecules is identical, an assumption that has been shown to be invalid (Baldi and Nasr, 2010).

In this study, we proposed and evaluated two parameter-free similarity search methods. The AS method considers information on the similarity to all query molecules, whereas the maxZ method converts the Tanimoto similarity into a z-score for a statistically sound, direct comparison. The results of our evaluations using over 20 HTS datasets indicated that neither method achieved significantly higher ROC_AUC values over the standard Max-Sim method. However, BEDROC values derived from both methods were ~10% higher than those of the Max-Sim method. Thus, although our methods perform comparably to the standard similarity search method when judged by ranking all compounds in a screening library, they perform better on early recognition by placing more actives at the top of a ranked list. This is an important trait for virtual screening of large chemical libraries, considering that follow-up experimental testing is feasible for only a small fraction of chemicals.

A conventional similarity search calculates TCs between all query molecules and all library molecules, and these values are sufficient for converting TCs to z-scores. As such, the additional computational cost to perform a similarity search using the maxZ method is minimal. Conversely, the AS method is notably slower than the Max-Sim method. However, with the ever-increasing power and decreasing cost of computing hardware, the method can become competitive based on its performance. Thus, when early recognition is among the objectives of virtual screening, the two methods provide alternatives to the standard Max-Sim method.

## Data Availability Statement

The raw data supporting the conclusions of this manuscript will be made available by the authors, without undue reservation, to any qualified researcher.

## Author Contributions

All authors contributed to the conception of the ideas and planned computational evaluations. RL performed the evaluations and wrote the first draft of the manuscript. AW supervised the project and contributed to the final manuscript.

### Conflict of Interest

The authors declare that the research was conducted in the absence of any commercial or financial relationships that could be construed as a potential conflict of interest.

## Footnotes

^1^https://www.cas.org/about/cas-content (accessed July 5, 2019).

^2^https://tripod.nih.gov/tox21/challenge/data.jsp (accessed July 5, 2019).

## References

[B1] Aherne G. W. McDonald E. Workman P. (2002). Finding the needle in the haystack: why high-throughput screening is good for your health. Breast Cancer Res. 4, 148–154. 10.1186/bcr440 12100740 PMC138735

[B2] Baldi P. Nasr R. (2010). When is chemical similarity significant? The statistical distribution of chemical similarity scores and its extreme values. J. Chem. Inf. Model. 50, 1205–1222. 10.1021/ci100010v 20540577 PMC2914517

[B3] Empereur-Mot C. Guillemain H. Latouche A. Zagury J. F. Viallon V. Montes M. (2015). Predictiveness curves in virtual screening. J. Cheminform. 7:52. 10.1186/s13321-015-0100-8 26539250 PMC4631717

[B4] Fontaine F. Bolton E. Borodina Y. Bryant S. H. (2007). Fast 3D shape screening of large chemical databases through alignment-recycling. Chem. Cent. J. 1:12. 10.1186/1752-153X-1-12 17880744 PMC1994057

[B5] Halgren T. A. Murphy R. B. Friesner R. A. Beard H. S. Frye L. L. Pollard W. T.. (2004). Glide: a new approach for rapid, accurate docking and scoring. 2. Enrichment factors in database screening. J. Med. Chem. 47, 1750–1759. 10.1021/jm030644s 15027866

[B6] Hawkins P. C. Skillman A. G. Warren G. L. Ellingson B. A. Stahl M. T. (2010). Conformer generation with OMEGA: algorithm and validation using high quality structures from the Protein Databank and Cambridge Structural Database. J. Chem. Inf. Model. 50, 572–584. 10.1021/ci100031x 20235588 PMC2859685

[B7] Huang N. Shoichet B. K. Irwin J. J. (2006). Benchmarking sets for molecular docking. J. Med. Chem. 49, 6789–6801. 10.1021/jm0608356 17154509 PMC3383317

[B8] Huang R. Xia M. (2017). Editorial: Tox21 Challenge to build predictive models of nuclear receptor and stress response pathways as mediated by exposure to environmental toxicants and drugs. Front. Environ. Sci. 5:3 10.3389/fenvs.2017.00003

[B9] Kairys V. Fernandes M. X. Gilson M. K. (2006). Screening drug-like compounds by docking to homology models: a systematic study. J. Chem. Inf. Model. 46, 365–379. 10.1021/ci050238c 16426071

[B10] Kar S. Roy K. (2013). How far can virtual screening take us in drug discovery? Expert Opin. Drug Discov. 8, 245–261. 10.1517/17460441.2013.761204 23330660

[B11] Kim S. Thiessen P. A. Bolton E. E. Chen J. Fu G. Gindulyte A.. (2016). PubChem substance and compound databases. Nucleic Acids Res. 44, D1202–D1213. 10.1093/nar/gkv951 26400175 PMC4702940

[B12] Kristensen T. G. Nielsen J. Pedersen C. N. (2013). Methods for similarity-based virtual screening. Comput. Struct. Biotechnol. J. 5:e201302009. 10.5936/csbj.201302009 24688702 PMC3962175

[B13] McClish D. K. (1989). Analyzing a portion of the ROC curve. Med. Decis. Making 9, 190–195. 10.1177/0272989X8900900307 2668680

[B14] Mysinger M. M. Carchia M. Irwin J. J. Shoichet B. K. (2012). Directory of useful decoys, enhanced (DUD-E): better ligands and decoys for better benchmarking. J. Med. Chem. 55, 6582–6594. 10.1021/jm300687e 22716043 PMC3405771

[B15] Nasr R. J. Swamidass S. J. Baldi P. F. (2009). Large scale study of multiple-molecule queries. J. Cheminform. 1:7. 10.1186/1758-2946-1-7 20298525 PMC3225883

[B16] Rogers D. Hahn M. (2010). Extended-connectivity fingerprints. J. Chem. Inf. Model. 50, 742–754. 10.1021/ci100050t 20426451

[B17] Sheridan R. P. Singh S. B. Fluder E. M. Kearsley S. K. (2001). Protocols for bridging the peptide to nonpeptide gap in topological similarity searches. J. Chem. Inf. Comput. Sci. 41, 1395–1406. 10.1021/ci0100144 11604041

[B18] Shiryaev S. A. Cheltsov A. V. Gawlik K. Ratnikov B. I. Strongin A. Y. (2011). Virtual ligand screening of the National Cancer Institute (NCI) compound library leads to the allosteric inhibitory scaffolds of the West Nile Virus NS3 proteinase. Assay Drug Dev. Technol. 9, 69–78. 10.1089/adt.2010.0309 21050032 PMC3033206

[B19] Triballeau N. Acher F. Brabet I. Pin J. P. Bertrand H. O. (2005). Virtual screening workflow development guided by the “receiver operating characteristic” curve approach. Application to high-throughput docking on metabotropic glutamate receptor subtype 4. J. Med. Chem. 48, 2534–2547. 10.1021/jm049092j 15801843

[B20] Truchon J. F. Bayly C. I. (2007). Evaluating virtual screening methods: good and bad metrics for the “early recognition” problem. J. Chem. Inf. Model. 47, 488–508. 10.1021/ci600426e 17288412

[B21] Zhao W. Hevener K. E. White S. W. Lee R. E. Boyett J. M. (2009). A statistical framework to evaluate virtual screening. BMC Bioinformatics 10:225. 10.1186/1471-2105-10-225 19619306 PMC2722655

